# Comparison of diagnostic accuracy of magnetic resonance elastography and Fibroscan for detecting liver fibrosis in chronic hepatitis B patients: A systematic review and meta-analysis

**DOI:** 10.1371/journal.pone.0186660

**Published:** 2017-11-06

**Authors:** Huanming Xiao, Meijie Shi, Yubao Xie, Xiaoling Chi

**Affiliations:** 1 Hepatology Department, Guangdong provincial hospital of Chinese Medicine, The Second Affiliated Hospital of Guangzhou University of Chinese Medicine, Guangzhou, Guangdong, China; 2 Guangzhou University of Chinese Medicine, Guangzhou, Guangdong, China; Universita degli Studi di Pisa, ITALY

## Abstract

**Aim:**

This systematic review and meta-analysis was carried out to compare the diagnostic accuracy of Magnetic resonance elastography (MRE) and Fibroscan for detecting liver fibrosis in Chronic Hepatitis B (CHB) patients.

**Methods:**

The PubMed, the Cochrane Library, and the Web of science databases were searched for studies that evaluated the diagnostic value of MRE and Fibroscan for liver fibrosis in CHB patients until March 1^st^ 2017. The quality of the included studies was assessed by the revised Quality Assessment for Studies of Diagnostic Accuracy tool (QUADAS-2). Meta-disc 4.1 was used to summary the area under receiver operating characteristics curve (AUROC), sensitivity, specificity, diagnostic odds ratios to assess the accuracy of staging liver fibrosis using MRE and Fibroscan.

**Results:**

A total of nine MRE studies with 1470 patients and fifteen Fibroscan studies with 3641 patients were included in this systematic review. The summary AUROC values using MRE and Fibroscan for detecting significant fibrosis, advanced fibrosis and cirrhosis were 0.981 vs. 0.796(p<0.001), 0.972 vs. 0.893(p<0.001), and 0.972 vs. 0.905 (p<0.001). The pooled sensitivity and specificity using MRE for the diagnosis of significant fibrosis, advanced fibrosis and cirrhosis were 92.8% and 93.7%, 89.6% and 93.2%, 89.5% and 92.0%, respectively. The pooled sensitivity and specificity using Fibroscan for the diagnosis of significant fibrosis, advanced fibrosis and cirrhosis were 71.6% and 81.6%, 79.0% and 84.6%, 80.0% and 86.6%, respectively.

**Conclusion:**

MRE is more accurate than Fibroscan in diagnosing liver fibrosis in CHB patients, especially in diagnosing significant fibrosis and advanced fibrosis.

## Introduction

Liver fibrosis is a vital wound-healing response during the process of progression from chronic hepatitis B (CHB) to cirrhosis[1]. It can be found in most of the CHB patients. Liver fibrosis progression can lead to cirrhosis, even result in long term sequelae, such as portal hypertension, liver failure, hepatocellular carcinoma and so on[1]. It is also considered to be the main cause of hepatitis B associated morbidity and mortality[2]. Hence, diagnosis of liver fibrosis plays an important role during the process of making therapeutic decisions as well as predicting disease outcomes, or following up of liver fibrosis progression in CHB patients.

Although liver biopsy has long been considered the “gold standard” for determine the stages of liver fibrosis, it cannot be widely used in clinical practice for its limitations and risks, such as sample errors, poor tolerance, high cost and risk of hemorrhage, etc. Therefore, an increasing number of investigations have focused on the noninvasive methods in order to more accurately identify patients with different stages of fibrosis[3–5]. Fibroscan (Transient Elastography) is one of such safer and more acceptable noninvasive models, which has been used widely in China and also been recommended by World Health Organization in clinical application[6]. Magnetic Resonance Elastography (MRE) is a new elastography method, which has been developed to improve the accuracy of diagnostic of liver fibrosis. However, the accuracy of MRE and Fibroscan is still controversial. Some researchers found that MRE has higher diagnostic accuracy than Fibroscan for staging liver fibrosis[7], while other researchers revealed that the accuracy of staging liver fibrosis is comparable between MRE and Fibroscan[8]. Therefore, the present meta-analysis aims to compare the diagnosis accuracy of MRE and Fibroscan for detecting liver fibrosis in CHB patients.

## Materials and methods

### Search strategy

The PubMed, the Cochrane Library, and the Web of Science databases were searched for studies, which evaluated the diagnostic value of MRE and Fibroscan for liver fibrosis in CHB patients until March 1^st^ 2017. The search terms included Magnetic resonance elastograpy, MR Elastography, Fibroscan, Transient elastography, liver biopsy, chronic hepatitis B, noninvasive models and liver fibrosis. The inclusion criteria of included studies was as follows: (1) the study evaluated the accuracy of MRE or Fibroscan for diagnosis liver fibrosis in CHB patients; (2) the study used liver biopsy as the reference standard for staging liver fibrosis; (3) the study reported sensitivity, specificity and the number of patients in different fibrosis stage which could be extracted to get the data of true positive, false positive, true negative and false negative; (4) the study enrolled more than 50 patients and (5) the studies should be published in international journals cited by SCI and the language should be in English. The studies with any of the following conditions were excluded: (1) studies were not relevant to MRE or Fibroscan diagnosis; (2) manuscripts have only abstracts, or correspondence letter, or author comments (3) animal studies or studies on children; (4) data incomplete or no liver biopsy or small sample size.

### Data extraction

Two reviewers extracted the required information of the included studies independently. The required data elements included author, region, the year of article publication, study design, patients’ age, sex, and liver biopsy scoring system, liver biopsy size, interval time between biopsy and MRE or Fibroscan as well as the sensitivity, specificity and the number of patients in different fibrosis stage. The quality of the studies included in this review was assessed by the revised Quality Assessment for Studies of Diagnostic Accuracy tool (QUADAS-2)[9]. Significant fibrosis, advanced fibrosis and cirrhosis was defined as stages of F2-F4, F3-F4 and F4 by Metavir score[10] or Batts and Ludwig score[11]. Liver stiffness assessed by Fibroscan is quantitatively analyzed for liver fibrosis and expressed as kilopascals (kPa).

### Data synthesis and analysis

Statistical analysis was performed by Meta-Disc software 1.4, Review Manager 5.3 and stata10.0. The data was extracted and the fourfold table was constructed to calculate the sensitivity, specificity, positive and negative likelihood ratio and diagnostic odds ratio (DOR). Area under the summary receiver operating characteristic (AUROC) curves and the cut-off were recalculated to compare the accuracy of MRE and Fibroscan for the diagnosis of significant fibrosis, advanced fibrosis and cirrhosis. A diagnostic tool is defined as perfect if AUC is 1.00, excellent if the AUC is greater than 0.90, good if it is greater than 0.80, moderate if it is less than 0.80[12]. The Z test was used to compare the summary AUROC values of these two noninvasive models for predicting liver fibrosis. The summary DORs, the summary sensitivity and specificity were also calculated to further examine the accuracy of MRE and Fibroscan for liver fibrosis.

### Assessment of heterogeneity and publication bias

The following methods have been used to evaluate the heterogeneity in our meta-analysis. The spearman’s correlation between the logit of sensitivity and 1-specificity was calculated to evaluate the threshold heterogeneity of the included studies. If P<0.05, it suggests that the threshold heterogeneity was observed. The Cochrane-Q test was used to assess the non-threshold heterogeneity of included studies. The inconsistency index I^2^ was calculated to qualify the amount of non-threshold heterogeneity. If the inconsistency index I^2^≥30%, ≥50% or ≥75% was considered as moderate, substantial or considerable heterogeneity, respectively. Possible publication bias was assessed by a linear regression test of funnel plot asymmetry using a Deeks plot.

## Results

### Search results

**Fig 1:**
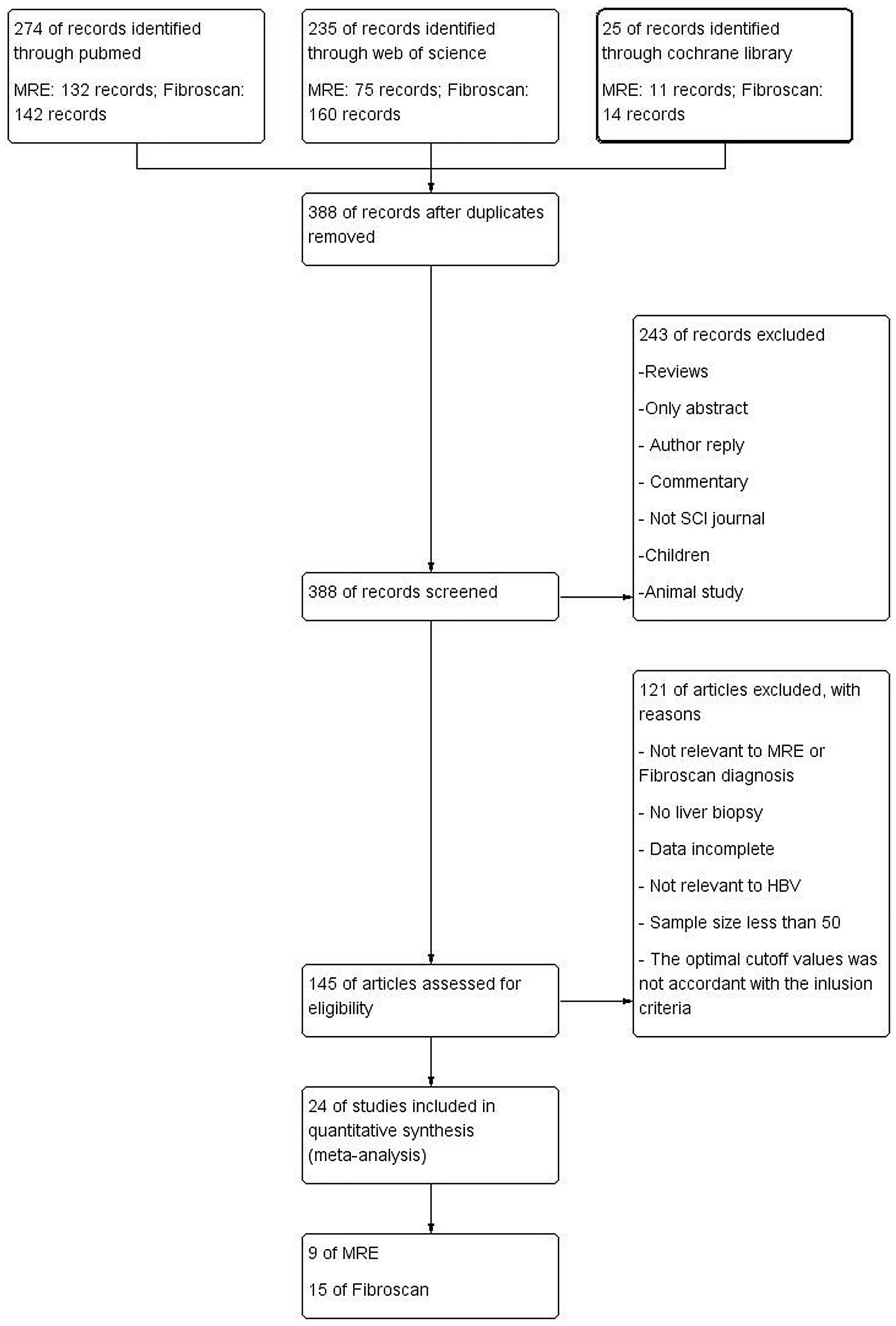
The study flow diagram. Published studies were identified to assess the accuracy of MRE and Fibroscan for diagnosing liver fibrosis.

388 studies were initially screened after removal of 146 duplicates. However, 363 studies were excluded for some reasons, such as only abstract, not relevant to MRE or Fibroscan diagnosis, animal study, or data incomplete, etc. Finally, 24 studies including 5111 patients were included for evaluation and meta-analysis[4, 7, 13–34]. The study flow diagram is shown in Fig 1.

### Basic characteristics of the included studies

**Fig 2:**
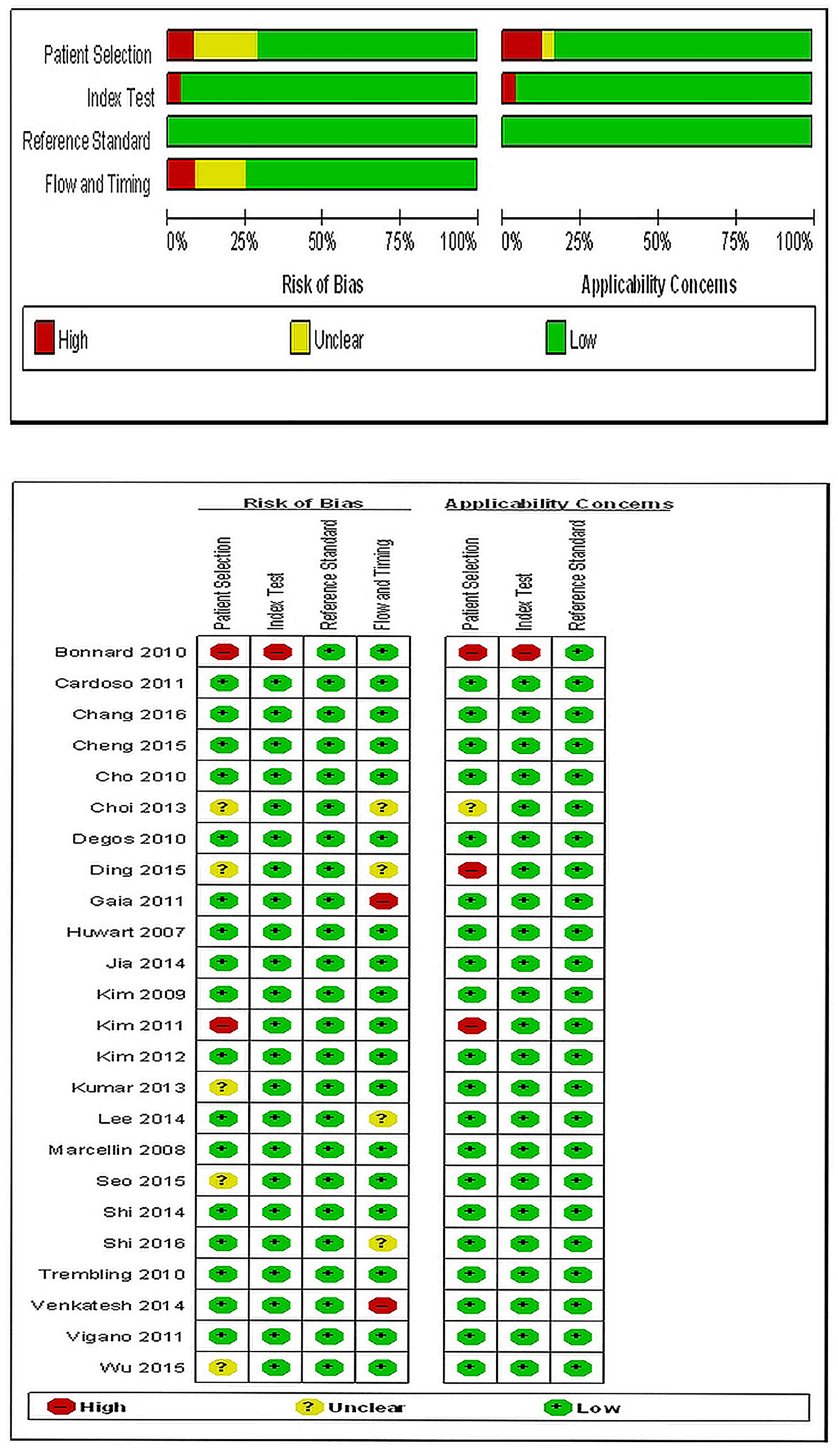
Methodological quality of included studies according to the revised quality assessment for studies of diagnostic accuracy tool (QUADAS-2) (+: yes; -: no; unclear).

Nine MRE studies including 1470 patients (mean age: 52.9 years; 65.0% male) and fifteen Fibroscan studies including 3641patients (mean age: 40.8 years; 72.3% male) were systematic reviewed. Twenty two studies used the Metavir score and two studies used the Batts and Ludwig score to assess the stages of liver fibrosis. Five of Nine MRE studies and thirteen of fourteen Fibroscan studies were prospective studies. More detailed characteristics of the enrolled studies were listed in Table 1, while Methodological quality of included studies according to QUADAS-2 was shown in Fig 2.

**Table 1:** Characteristics of the included studies.

Models	Author, Year,Region	Study/center Description	N	Interval between biopsy and MRE or Fibroscan	Median/MeanAge(Years)Males (%)	Liver biopsy scoring system	Liver Biopsy length (mm)	For diagnosis stages of liver fibrosis
**Fibroscan**	Bonnard, 2010,France	Prospective One center	59	<6 months	35(68)	METAVIR	Unclear	F2, F4
**Fibroscan**	Cardoso, 2012,France	ProspectiveOne center	202	Same day	41(80)	METAVIR	19±6	F2, F3,F4
**Fibroscan**	Cheng, 2015,China	ProspectiveSeven enters	459	Same day	33(74)	METAVIR	>10	F2, F3
**Fibroscan**	Cho, 2011,Korea	ProspectiveOne center	121	Same day	39(67)	Batts and Ludwig	>15	F2, F3
**Fibroscan**	Gaia, 2011,Italy	ProspectiveOne center	70	<6 months	44(71)	METAVIR	>20	F2, F3
**Fibroscan**	Jia, 2015,China	ProspectiveSeven centers	469	<6 months	34(74)	METAVIR	>10	F2, F3
**Fibroscan**	Kim, 2012,Korea	ProspectiveOne center	194	Same day	47(61)	Batts and Ludwig	>20	F2, F3,F4
**Fibroscan**	Marcellin, 2009,France	ProspectiveFive centers	173	<3 months	40(67)	METAVIR	16±6	F2, F3,F4
**Fibroscan**	Seo, 2015,Korea	ProspectiveFifty-five centers	567	<3 months	45(67)	METAVIR	>15	F2, F3,F4
**Fibroscan**	Degos, 2010, France	Prospectivetwenty-three centers	284	<1month	38(81)	METAVIR	24	F4
**Fibroscan**	Ding, 2015,China	RetrospectiveOne center	406	Unclear	42(73)	METAVIR	unclear	F4
**Fibroscan**	Kumar,2013,India	ProspectiveOne center	200	<1week	38(80)	METAVIR	>15	F4
**Fibroscan**	Trembling,2014,Italy	ProspectiveOne center	182	Same day	46(71)	METAVIR	>20	F3, F4
**Fibroscan**	Vigano, 2011, Italy	ProspectiveOne center	125	Same day	47(71)	METAVIR	>20	F4
**Fibroscan**	Kim, 2009, Korea	ProspectiveOne center	130	Same day	43(79)	METAVIR	Unclear	F4
**MRE**	Chang, 2016, Korea	RetrospectiveOne center	281	<3 months	56(57)	METAVIR	>20	F2, F3,F4
**MRE**	Choi, 2013, Korea	RetrospectiveOne center	173	Unclear	57(75)	METAVIR	Unclear	F2, F3,F4
**MRE**	Huwart, 2008, Belgium	ProspectiveOne center	88	2days	54(42)	METAVIR	34±10	F2, F3,F4
**MRE**	Kim, 2011, Korea	ProspectiveOne center	60	<58 days	58(84)	METAVIR	Unclear	F2, F3,F4
**MRE**	Lee, 2014, Korea	RetrospectiveOne center	334	Unclear	56(81)	METAVIR	Unclear	F2, F3,F4
**MRE**	Shi, 2014,China	ProspectiveOne center	113	23days	42 (43)	METAVIR	14±7	F2, F3,F4
**MRE**	Shi, 2016, China	ProspectiveOne center	173	Unclear	43(60)	METAVIR	>15	F2, F3,F4
**MRE**	Venkatesh, 2014, Singapore	ProspectiveOne center	63	<6months	50(70)	METAVIR	Unclear	F2, F3,F4
**MRE**	Wu, 2015, Taiwan	RetrospectiveOne center	185	Unclear	59(75)	METAVIR	>10	F2, F3,F4

### Results of meta-analysis

#### Diagnostic accuracy of MRE and Fibroscan for the prediction of significant fibrosis

**Fig 3:**
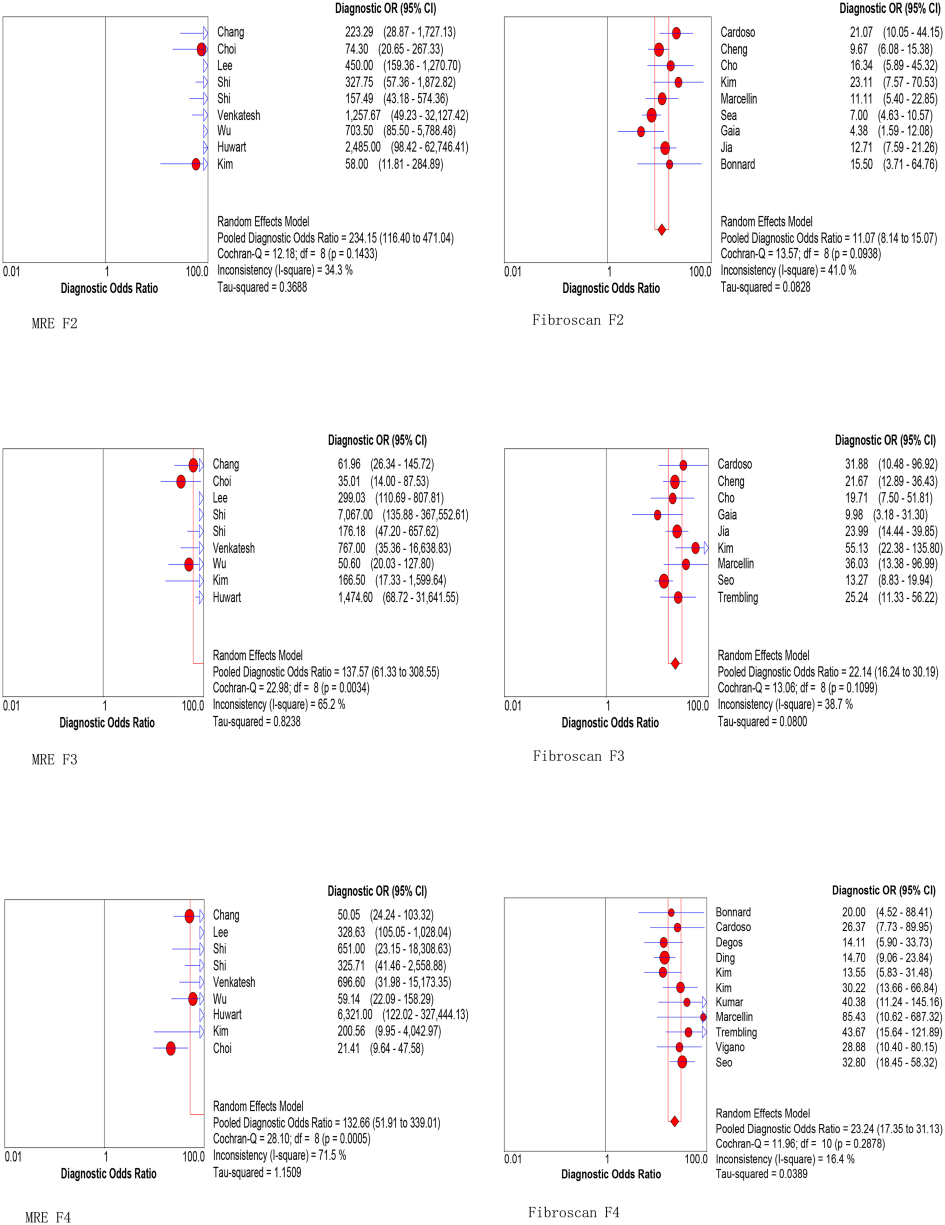
Forest plots of the DOR of MRE and Fibroscan for staging significant fibrosis (F2), advanced fibrosis (F3) and cirrhosis (F4). The summary DOR of MRE is superior to Fibroscan for detecting significant fibrosis (F2), advanced fibrosis (F3) and cirrhosis (F4).

**Fig 4:**
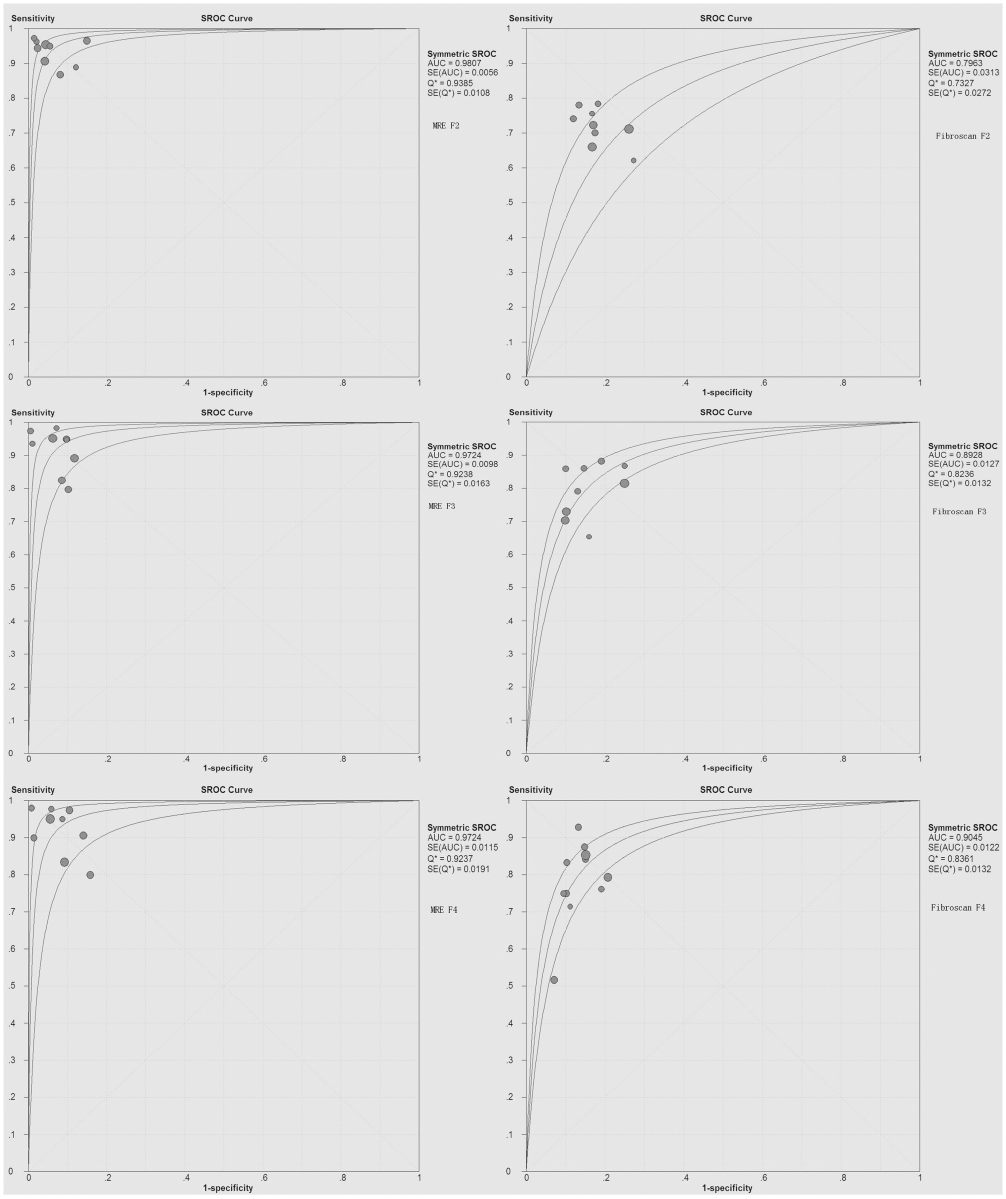
Summary ROC curve of the diagnostic accuracy of MRE and Fibroscan for staging significant fibrosis (F2), advanced fibrosis (F3) and cirrhosis (F4). The summary AUROC values using MRE for detecting significant fibrosis(F2), advanced fibrosis(F3) and cirrhosis(F4) were all significantly higher than that using Fibroscan.

Nine MRE studies including 1470 patients and Nine Fibroscan studies including 2314 patients were enrolled for diagnosis of significant fibrosis. As is shown in Table 2, both the pooled sensitivity and specificity using MRE (92.8% and 93.7%) with a cutoff value of 2.99 kPa is higher than using Fibroscan for significant fibrosis (71.6%and 81.6%) with a cutoff value of 7.53 kPa. Furthermore, the summary DOR of MRE is superior to Fibroscan (234.15vs11.07, Fig 3 MRE F2 vs. Fibroscan F2). More importantly, the summary AUROC values using MRE for detecting significant fibrosis was significantly higher than that using Fibroscan (0.981 vs. 0.794, P<0.001, Fig 4).

**Table 2:** Meta-analysis results of MRE and Fibroscan for prediction of significant fibrosis, advanced fibrosis and cirrhosis.

	Number of Studies (Patients)	Cutoff value (Mean, Range) (kPa)	Summary Sensitivity (95%CI, %)	Summary Specificity (95%CI, %)	Summary LR+ (95%CI)	Summary LR- (95%CI)	Summary AUROC	Summary DOR(95%CI)
**Significant fibrosis**								
**MRE**	9 (2314)	2.99 (2.5–4.69)	92.8 (91.0–94.4)	93.7 (91.3–95.6)	14.27 (8.14–25.01)	0.07 (0.05–0.11)	0.981	234.15 (116.40–471.04)
**Fibroscan**	9 (1470)	7.53 (7.2–8.5)	71.6 (69.1–74.0)	81.6 (78.9–84.2)	3.83 (3.11–4.73)	0.35 (0.31–0.39)	0.796	11.07 (8.14–15.07)
**Advanced fibrosis**								
**MRE**	9 (1470)	3.62 (2.92–5.45)	89.6 (87.1–91.7)	93.2 (91.1–94.9)	10.87 (7.92–14.89)	0.09 (0.06–0.16)	0.972	137.57 (61.33–308.55)
**Fibroscan**	9 (2437)	9.15 (8.1–10.5)	79.0 (76.1–81.6)	84.6 (82.7–86.3)	5.23 (4.02–6.79)	0.25 (0.20–0.31)	0.893	22.15 (16.24–30.19)
**Cirrhosis**								
**MRE**	9 (1470)	4.63 (3.67–4.87)	89.5 (86.2–92.2)	92.0 (90.2–93.6)	10.74 (7.12–16.21)	0.10 (0.05–0.18)	0.972	132.66 (51.91–339.01)
**Fibroscan**	11 (2522)	12.17 (11–14)	80.0 (76.7–83.0)	86.6 (85.0–88.1)	5.81 (4.89–6.90)	0.25 (0.194–0.328)	0.905	23.24 (17.35–31.13)

#### Diagnostic accuracy of MRE and Fibroscan for the prediction of advanced fibrosis

Nine MRE studies including 1470 patients and Nine Fibroscan studies including 2437 patients were enrolled for diagnosis of advanced fibrosis. As is shown in Table 2, both the pooled sensitivity and specificity using MRE (89.6% and 93.2%) with a cutoff value of 3.62 kPa is higher than using Fibroscan for advanced fibrosis (79.0%and 84.6%) with a cutoff value of 9.15 kPa. Furthermore, the summary DOR of MRE is superior to Fibroscan (137.57 vs. 22.15, Fig 3 MRE F3 vs Fibroscan F3). More importantly, the summary AUROC value using MRE for detecting advanced fibrosis was significantly higher than that using Fibroscan (0.972 vs. 0.893, p<0.001, Fig 4).

#### Diagnostic accuracy of MRE and Fibroscan for the prediction of cirrhosis

Nine MRE studies including 1470 patients and eleven Fibroscan studies including 2522 patients were enrolled for diagnosis of cirrhosis. As is shown in Table 2, both the pooled sensitivity and specificity using MRE (89.5% and 92.0%) with a cutoff value of 4.63 kPa is higher than using Fibroscan for cirrhosis (80.0%and 86.6%) with a cutoff value of 12.17 kPa. Furthermore, the summary DOR of MRE is superior to Fibroscan (132.66 vs. 23.24, Fig 3 MRE F4 vs. Fibroscan F4). More importantly, the summary AUROC value using MRE for detecting cirrhosis was significantly higher than that using Fibroscan (0.972 vs. 0.905, p<0.001, Fig 4).

### Methodological heterogeneity and publication bias

**Fig 5:**
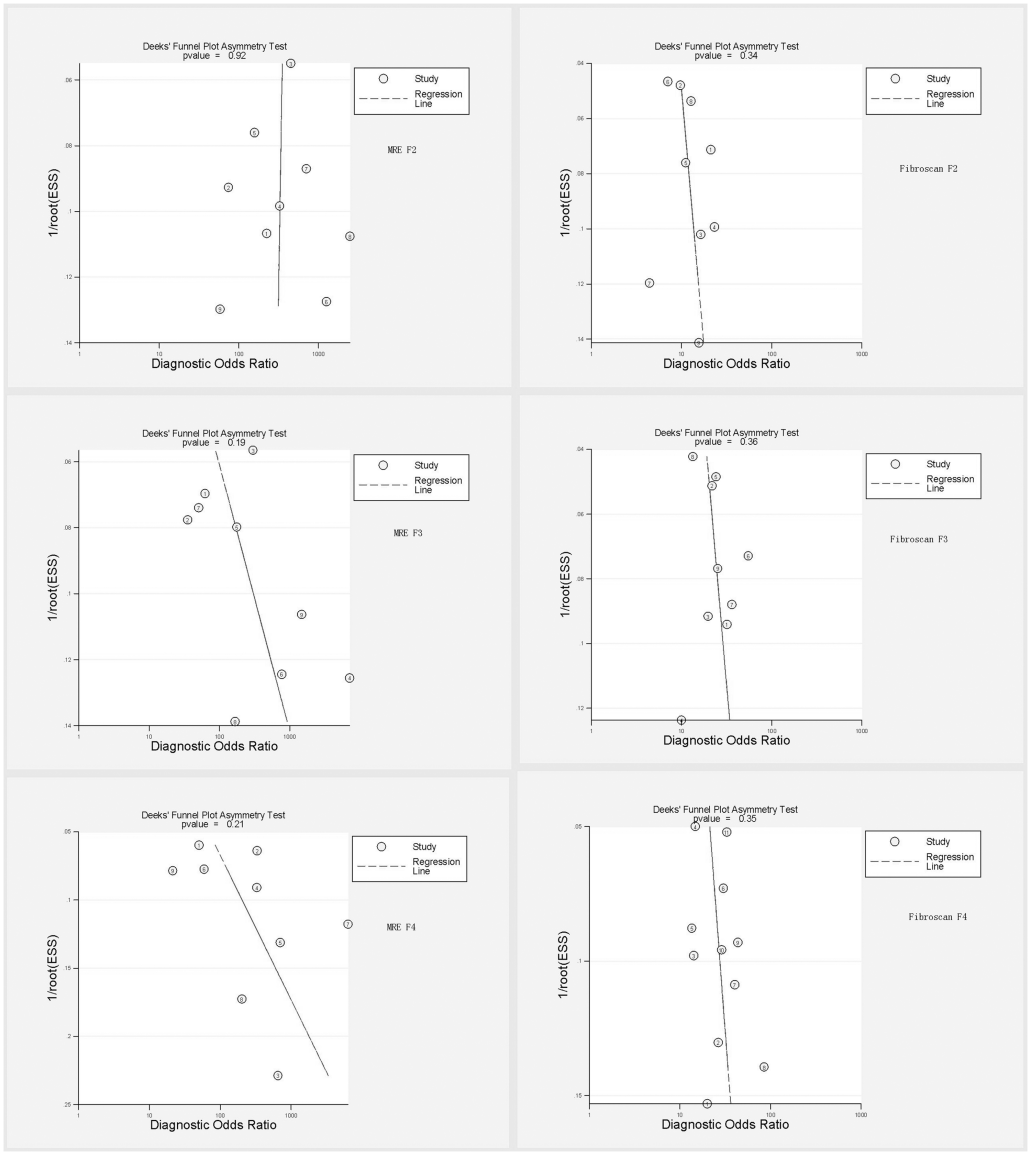
Deek’s funnel plot asymmetry test for publication bias. Considerable heterogeneity and publication bias was not observed in both MRE and Fibroscan studies for detecting liver fibrosis in CHB patients.

Threshold heterogeneity was not observed in both MRE and Fibroscan studies. Non-threshold heterogeneity was observed in some groups, especially for MRE F3 and MRE F4 with substantial heterogeneities (I^2^ = 65.2% and 71.5%, respectively) (Table 3). Considerable heterogeneity and publication bias was not observed in both MRE and Fibroscan studies for detecting significant fibrosis, advanced fibrosis and cirrhosis in CHB patients (Fig 5).

**Table 3:** Heterogeneity of all the included studies.

Fibrosis stage	Threshold heterogeneity		Non-Threshold heterogeneity	
	r	p value	I^2^(%)	p
**MRE**				
**F2**	-0.383	0.308	34.3	0.143
**F3**	-0.600	0.088	65.2	0.003
**F4**	-0.550	0.125	71.5	<0.001
**Fibroscan**				
**F2**	-0.450	0.224	41.0	0.094
**F3**	0.500	0.170	38.7	0.110
**F4**	0.524	0.098	16.4	0.288

## Discussion

Due to the controversial accuracy of MRE and Fibroscan for staging liver fibrosis, this current meta-analysis summarized the diagnosis accuracy of MRE and Fibroscan for detecting liver fibrosis in CHB patients. Finally, a total of nine MRE studies and fifteen Fibroscan studies were systematic reviewed. The results demonstrate that MRE had an excellent diagnostic accuracy for detecting liver fibrosis with a summary AUROC of 0.981, 0.972 and 0.972 for significant fibrosis, advanced fibrosis and cirrhosis, respectively, while Fibroscan had a moderate to excellent accuracy with a summary AUROC of 0.796, 0.893 and 0.905 respectively. MRE showed summary AUROC greater than 90% for detecting different stages of liver fibrosis in CHB patients. This is comparable to the results of the previous meta-analysis[35] (0.97, 0.96 and 0.97) which included patients with different etiologies. However, the summary AUROC values for Fibroscan detecting significant fibrosis was less than 80% in our meta-analysis, which was lower than the previous studies’ results [36, 37]. In Chon’s and Li’s meta-analysis[5, 30], the mean AUROC for diagnosis significant fibrosis was 0.859 and 0.88. The sensitivity of Fibroscan for significant fibrosis in our review was also lower than that of Li’s review (0.716 vs. 0.806). It might due to the reason that we reviewed the Fibroscan studies with similar liver stiff cutoff values recommended by WHO guidelines[6]. Therefore, the heterogeneity is lower than previous meta-analysis of Fibroscan for detecting liver fibrosis. Based on these results of our meta-analysis, we claim that both Fibroscan and MRE are excellent tools for diagnosis cirrhosis in CHB patients. However, MRE is more accurate than Fibroscan for detecting significant fibrosis and advanced fibrosis. Compared to shear wave elastography (SWE) reported in another meta-analysis[38], MRE showed greater summary AUROC for detecting significant fibrosis (0.98 vs 0.88). However, the diagnostic accuracy using SWE and MRE is comparable when detecting advanced fibrosis (sAUROC 0.972 vs 0.94) and cirrhosis (sAUROC 0.972 vs 0.92). Therefore, MRE might be used more popular as a noninvasive tool for detecting significant fibrosis in CHB patients.

The mean optimal cutoff values of MRE in our study were 2.99 for significant fibrosis, 3.62 for advanced fibrosis and 4.63 for cirrhosis. These cutoff values of MRE were lower than that of Singh’s study[39] (3.66, 4.11 and 4.71) with 47.1% chronic hepatitis C patients. It may due to the different etiology in these two studies. Concerning to Fibroscan, the mean optimal cutoff values were 7.53, 9.15 and 12.17 for significant fibrosis, advanced fibrosis and cirrhosis, respectively. These were also lower than the studies in chronic hepatitis C (CHC) patients. For example, Stebbing et al[40] claimed higher cutoff values (8.44 kPa and 16.14 kPa for significant fibrosis and cirrhosis) when calculated only in CHC patients. This tendency of low cutoff values of Fibroscan in CHB patients and high cutoff values in CHC is similar to the results of Chon’s study[5]. Sturm’s[41] conclusion might explain this tendency. He claimed that the fibrous septa might be thinner in CHB patients than in CHC patients with the same liver fibrosis stage so that the total amount of liver fibrosis reflected by the fibrosis area was significantly lower in CHB patients.

There are some limitations in our study. Firstly, as MRE is a new elastography method, the number of MRE studies is so limited that we could not set more stringent inclusion criteria. For example, just enrolled prospective studies or enrolled only studies with clear information of the liver biopsy tissue length, or even enrolled studies with the similar distribution of the enrolled studies. Due to these reasons, non-threshold substantial heterogeneity was observed in MRE F3 and MRE F4 groups. Thus, large-scale, well-designed, and multi-center studies are needed to validate the conclusion and further evaluate the potential of MRE. Secondly, there are only a few studies focus on the direct comparison between MRE and Fibroscan. Hence, further large prospective direct comparison studies of MRE and Fibroscan should be conducted to confirm the high accuracy of MRE in CHB patients. Finally, only SCI articles in English were selected for meta-analysis, this language and SCI restriction might bias the results to some extent.

## Conclusions

In summary, MRE is more accurate than Fibroscan in diagnosing liver fibrosis in CHB patients, especially in diagnosing significant fibrosis and advanced fibrosis. Although Fibroscan had a moderate accuracy in diagnosis significant fibrosis and advanced fibrosis, it is more cheaper and more easily popular used in clinical practice. Future studies on this issue should focus on standardization of the parameters for both imaging modalities to make them more feasible in clinical practice.

## Supporting information

S1 PRISMA Checklist PRISMA 2009 checklist. (DOC)
